# Deep learning reveals enhanced ENSO predictability under historical anthropogenic forcing

**DOI:** 10.1126/sciadv.aec9518

**Published:** 2026-06-10

**Authors:** Zikuan Lin, Yishuai Jin, Chengfei He, Shaoqing Zhang, Michael J. McPhaden, Xiaopei Lin

**Affiliations:** ^1^ State Key Laboratory of Physical Oceanography/Physical Oceanography Laboratory, Ocean University of China, Qingdao, China.; ^2^ Academy of Future Ocean, Ocean University of China, Qingdao, China.; ^3^ SANYA Oceanographic Laboratory, Sanya, China.; ^4^ Department of Marine and Environmental Sciences, Marine Science Center, Northeastern University, Nahant, MA, USA.; ^5^ Pacific Marine Environmental Laboratory, NOAA, Seattle, WA, USA.

## Abstract

Understanding how ENSO predictability responds to climate change is essential for improving future climate projections. In this study, we apply a deep learning model—convolutional neural network with a leave-one-out model strategy to Coupled Model Intercomparison Project Phase 6 (CMIP6) historical and preindustrial control simulations. We find that El Niño–Southern Oscillation (ENSO) predictability is statistically enhanced by 14.0 ± 1.8% enhancement under historical anthropogenic forcing. This improvement is linked to changes in key ocean-atmosphere feedbacks. Under historical forcing, the equatorial Pacific shows a shoaling of the thermocline. As a result, the surface ocean becomes more sensitive to wind forcing, and subsurface temperature becomes more responsive to thermocline variations. These processes strengthen the three-dimensional advective and thermocline feedbacks, which together enhance ENSO predictability. These findings highlight the importance of anthropogenic forcing in shaping ENSO predictability in a warming climate.

## Teaser

Deep learning has revealed that ENSO predictability has improved by 14.0% under historical forcing.

## INTRODUCTION

The El Niño–Southern Oscillation (ENSO), a coupled ocean-atmosphere phenomenon originating in the tropical Pacific, is the most important driver of year-to-year climate variations on Earth (*1*, *2*). The warm and cold phases of ENSO alter global atmospheric circulation and patterns of weather variability via planetary-wave teleconnections, triggering climate extremes worldwide (*3*, *4*). Given its far-reaching consequences, recent studies have examined how the behavior of ENSO has changed under anthropogenic historical forcing, suggesting that ENSO-related sea surface temperature (SST) variability has increased since the 1960s, characterized by more frequent and intense El Niño and La Niña events (*5*, *6*). These changes are caused by intensified upper-ocean stratification in the equatorial Pacific, which strengthens ocean-atmosphere coupling and amplifies the response of surface mixed-layer to atmospheric forcing (*6*, *7*).

Compared to the basic characteristics of ENSO, investigations of ENSO predictability under the historical forcing have been rare. Previous studies have shown analytically and numerically that ENSO predictability is related to the growth rate of ENSO and the delayed thermocline (TH) response under recharge oscillator framework (*8*). Specifically, a larger ENSO growth rate and a stronger delayed TH response contribute to a higher ENSO predictability. By using this analytical solution and 36 dynamical models in Coupled Model Intercomparison Project Phase 6 (CMIP6) under shared socioeconomic pathways (SSP5-8.5) scenarios, some studies suggested a lower ENSO growth rate caused by a stronger thermodynamical damping in the future, leading to weakened ENSO predictability (*9*). However, it remains unknown how historical forcing affects ENSO predictability compared with preindustrial forcing. It is possible that the TH feedback is strong and the thermodynamical damping is weak under historical forcing, which may lead to different conclusions about changes in ENSO predictability compared to those under SSP5-8.5 scenarios.

Recent advances in deep learning have improved our ability to predict and understand ENSO. These efforts include forecasts of ENSO-related Niño index (*10*, *11*) and spatiotemporal predictions of SST fields (*12*), achieved by leveraging advanced architecture such as transformer (*13*) or integrating dynamical model outputs (*14*). As a result, they enable skillful predictions up to 17 months in advance, far exceeding the 6-month lead time of the linear inverse model (*15*), while offering higher computational efficiency than conventional dynamical models. This breakthrough makes deep learning model a powerful tool for assessing ENSO predictability (*16*).

Here, we train a convolutional neural network (CNN) prediction model with data from the latest climate models participating in the CMIP6. In this way, we quantify a robust 14.0±1.8% enhancement of ENSO predictability under historical forcing compared with preindustrial forcing. Further analysis within the recharge oscillator model (ROM) (*17*, *18*) framework shows that the intensified ocean stratification can increase the ENSO growth rate, thus enhancing its predictability (*19*).

## RESULTS

### Enhanced ENSO predictability under historical forcing revealed by CNN

**Fig. 1.**
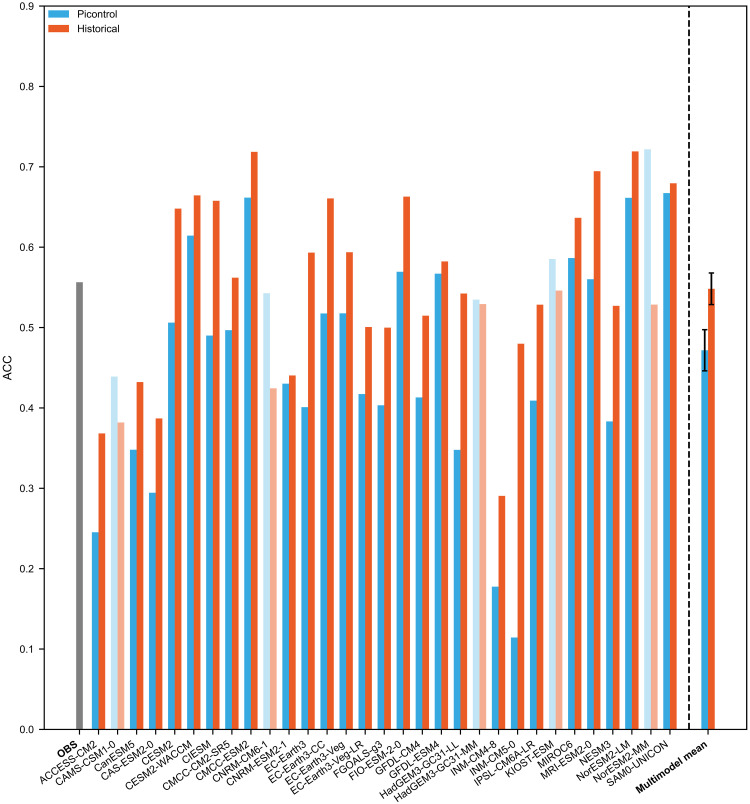
CNN based estimation of ENSO predictability under preindustrial and historical forcing. Average prediction skill of the CNN model, expressed as the ACC over lead times from 1 to 24 months, under preindustrial (blue bars) and historical (orange bars) forcing. Error bars indicate 1 SD, computed via bootstrap resampling method. Models showing a trend opposite to the multimodel mean are displayed in light colors.

We assess ENSO predictability across 31 CMIP6 models using a CNN model within the leave-one-out model framework (see Materials and Methods). Since our predictand is the Niño3.4 index, we excluded three models in CMIP6 whose simulated El Niño peak phase deviate notably from observations (fig. S1). We choose a CNN deep learning model because it achieves competitive prediction skill compared to the state-of-the-art ENSO prediction models (*13*) while maintaining low computational cost. This efficiency makes it feasible to train and evaluate the model across 31 individual CMIP6 models. Our experiment reveals a robust enhancement in mean anomaly correlation coefficient (ACC) skill for ENSO prediction when comparing the historical forcing to preindustrial simulations (Fig. 1). Specifically, 83.9% of the models (26 of 31) exhibit improved prediction skill under historical forcing, indicating a strong model consensus on the enhanced predictability. The multimodel mean ACC increases by 14.0%, and this improvement is statistically significant at 95% confidence level based on the bootstrap method. Our results remain robust when averaged across different lead-time ranges (fig. S2) and different model selection strategies (figs. S3 and S4; see Materials and Methods for details). In addition to the ACC, we also assess the spring predictability barrier (SPB) strength, as defined in Materials and Methods, which provides another metric for evaluating ENSO predictability (*9*). Using two different definitions of SPB strength, both metrics show notable reduced SPB strength under historical forcing, with 64.5 and 74.2% of models (20 of 31 and 23 of 31, respectively) exhibiting weaker SPB. Consistent with the overall enhancement in ENSO predictability, the multimodel mean SPB strength decreases by 10.2 and 17.6%, respectively, with both reductions statistically significant at the 95% confidence level (fig. S5).

### Understanding ENSO predictability with ROM

**Fig. 2.**
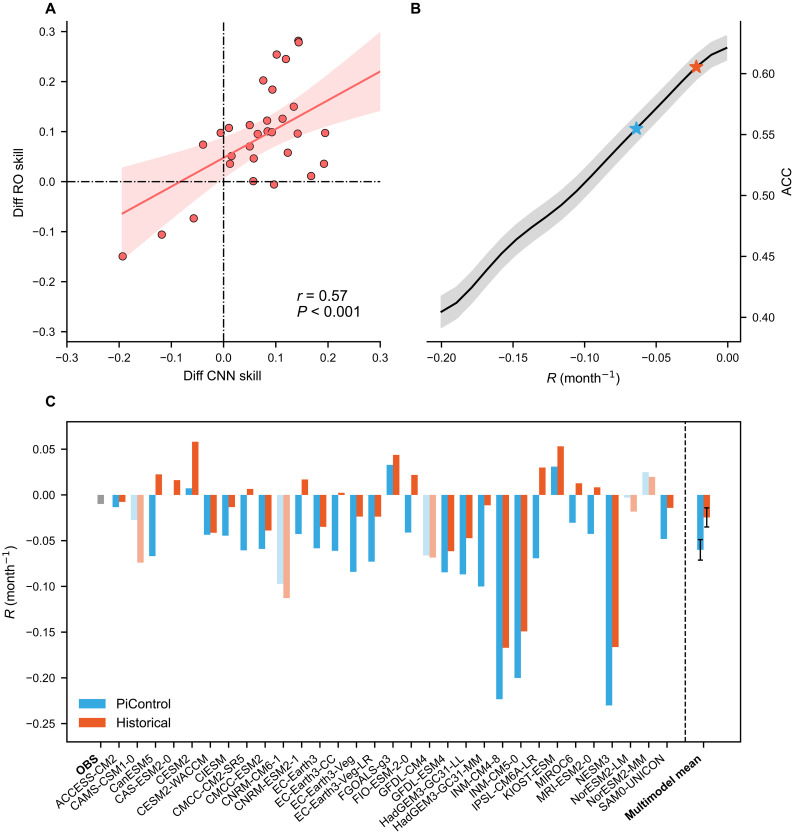
Recharge oscillator framework explains enhanced ENSO predictability under historical forcing. (**A**) Intermodel relationship between changes in CNN and ROM prediction skill (historical minus preindustrial), with shading indicating the 95% confidence interval. ROM skill is averaged over 1- to 12-month lead times, while CNN skill is averaged over 1 to 24 months. See Materials and Methods for details. (**B**) The prediction skill of ROM across the parameter space of ENSO growth rate *R*, averaged over lead times of 1 to 12 months. Star markers indicate the ensemble-mean values of *R* for preindustrial (blue) and historical (orange) forcings, as quantified in (C). (**C**) Intermodel comparison of *R* across CMIP6 models under preindustrial (blue bars) and historical (orange bars) forcings. The shading in (B) and error bars in (C) denote 1 SD, computed via bootstrap resampling method (see Materials and Methods).

To understand the factors underlying the change of ENSO predictability, we use the ROM framework (see Materials and Methods). The simplified conceptual model captures the essential physics of ENSO dynamics and allows us to isolate the roles of individual parameters that govern its behavior. For each CMIP6 model, we estimate the ROM parameters by directly fitting the simulated SST anomalies (SSTAs) and sea surface height anomalies (SSHAs). We first predict the Niño 3.4 index with the fitted ROM (see Materials and Methods for details). Despite the lower skill of ROM compared to CNN model, we find a notable correlation between the skill changes of ROM and CNN under different forcings (Fig. 2A). This suggests that the ROM can be used to provide a physically interpretable framework for diagnosing the physical mechanisms behind changes in ENSO predictability.

To identify the influence of ROM parameters on its predictability, we conduct a series of controlled numerical experiments. We begin with a set of perfect model experiments, in which the forecast model is identical to the model used to generate the synthetic truth, and the only source of uncertainty arises from small perturbations in initial conditions (see Materials and Methods for detailed setting). By varying each ROM parameter individually while holding others fixed, we isolate their influence on predictability (Fig. 2B and fig. S6). The results show a clear pattern: Increasing *R*, $F_{1}$, and $F_{2}$ leads to higher prediction skill, while larger ε reduces predictability. Here, R is the growth rate of the SSTA, $F_{1}$ and $F_{2}$ measure the strength of key feedbacks between SST and TH depth, and ε indicates the damping rate of the TH itself.

Among the four parameters in ROM, we find that the growth rate *R* emerges as the dominant contributor to predictability changes across historical and preindustrial forcings. An intermodel comparison reveals that *R* increases by 59.1% on average under historical forcing relative to preindustrial control, with 26 of 31 models (83.9%) showing consistent increases (Fig. 2C). In contrast, the other ROM parameters, including $F_{1}$, $F_{2}$, and ε, do not exhibit consistent changes across models (fig. S7). To quantify the impact of this change, we evaluate ROM-based prediction skill under both forcing scenarios using their respective parameter sets. The historical runs consistently yield higher prediction skill across different lead times (fig. S8). To further assess this result, we conduct an “imperfect model experiment,” in which we forecast historical events using R estimated from the preindustrial runs (see Materials and Methods). Despite this mismatch, we still observe higher prediction skill in the historical period (fig. S9), indicating that inherent changes in ENSO characteristics under historical forcing play a key role in enhancing predictability. This behavior has been reported previously and is commonly referred to as the skill-persistence rule (*20*), whereby increased system persistence can lead to improved forecast skill even in the presence of model imperfections. These results not only confirm that the increased growth rate under historical forcing contributes robustly to enhanced ENSO predictability but also support the credibility of our CNN-based prediction results.

### Physical interpretation of strengthened ENSO predictability under historical forcing

To further investigate the physical drivers behind the enhanced ENSO predictability under historical forcing, we diagnose the ENSO growth rate based on a heat budget analysis. This growth rate, also known as Bjerknes (BJ) stability index, can be decomposed into several key dynamic and thermodynamic feedback components (see Materials and Methods). To avoid confusion with the SSTA-SSHA regression–based estimate of *R*, we refer this metric as BJ index hereafter. We quantified the contribution of each feedback to the change in this index from preindustrial to historical forcing. Overall, the BJ indices are consistent with the SSTA-SSHA regression–based estimates, with both showing an increase. Specifically, the BJ index increases about 69.4% under historical forcing compared with preindustrial forcing, with high model consensus (89.5%, 17 of 19 models) (fig. S10B). Note here that we only use 19 models to calculate BJ index because some ocean variables are not available in all the models (see table S1). In general, historical forcing enhances both four positive feedbacks of TH feedback, meridional advective (MA) feedback, vertical advective (VA) feedback, and zonal advective (ZA) feedbacks, and two negative feedbacks of thermodynamical damping (TD) feedbacks, and dynamical damping (DD) feedbacks (fig. S10A).

**Fig. 3.**
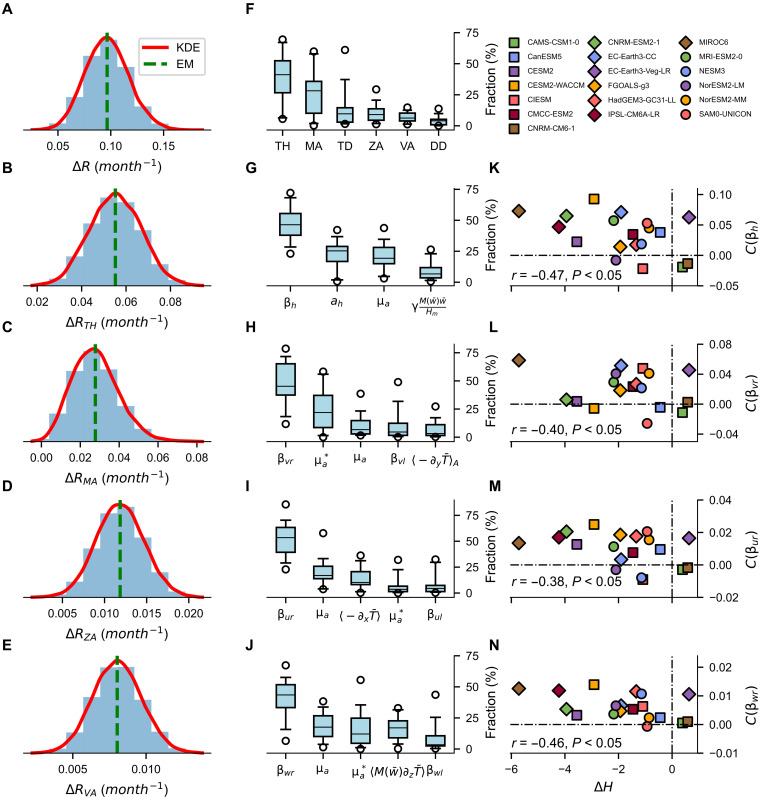
Physical drivers behind changes in ENSO growth rate under preindustrial and historical forcings. (**A**) Distribution of changes in ENSO growth rate (historical minus preindustrial), estimated via bootstrap resampling methods. The red line indicates the kernel density estimation using Gaussian kernels, the green line denotes the ensemble mean. (**B** to **E**) The same as (A) but for changes in (B) TH feedback (TH), (C) MA feedback, (D) ZA feedback, and (E) VA feedback. (**F**) Boxplot of the fractional contribution of individual feedback to changes in ENSO growth rate. Boxes represent the interquartile range (from first to the third quantile), whiskers indicate the 5 and 95% percentiles, and outliers are shown as individual points. (**G** to **J**) The same as (F) but for the fractional contributions of different parameters to (G) TH, (H) MA, (I) ZA, and (J) VA. (**K** to **N**) Changes in TH depth (Δ*H*) versus changes in the contribution of (K) zonal SSHA slope response to central equatorial wind anomalies (β_h_) to TH, (L) meridional current response to central equatorial wind anomalies (β_vr_) to MA, (M) zonal current response to central equatorial wind anomalies (β_ur_) to ZA, and (N) vertical current response to central equatorial wind anomalies (β_wr_) to VA.

To quantitively assess the relative importance of each contributing factor, we calculate their fractional contribution (see Materials and Methods). Here, we focus on the positive feedbacks, which are the primary contributor to the increase of ENSO growth rate under historical forcing, as summarized in Fig. 3. From Fig. 3F, the increase in ENSO growth rate is primarily driven by TH and MA, accounts for 41.1±6.6% and 28.2±6.0% of total changes, respectively. The ZA and VA make relatively smaller contributions, accounts for 9.0±2.5% and 6.2±1.5% changes, respectively. All the confidence interval is estimated using bootstrap resampling method.

The amplification of TH can be further decomposed into contributions from its underlying physical parameters. Specifically, Fig. 3G shows that the increase in TH is dominated by a stronger response of zonal SSHA slope to central equatorial ($5^{\circ }S-5^{\circ }N,150^{\circ }E-130^{\circ }W$) wind forcing ($\beta_{h}$). On average, changes in $\beta_{h}$ contribute 46.4±3.1% of the total change in TH. This result is consistent with an empirical study of ENSO decadal variability using reanalysis products in which the zonal slope of the TH was the dominant factor controlling the TH feedback and the BJ index variations (*21*). Similarly, the increased strength of MA is primarily associated with the increased sensitivity of meridional current to the wind forcing, and the same mechanism applies to ZA and VA. As shown in Fig. 3 (H to J), the changes in $\beta_{\mathrm{vr}}$, $\beta_{\mathrm{ur}}$, and $\beta_{\mathrm{wr}}$, which represent the responses of meridional, zonal, and vertical ocean currents to wind forcing, account for most of the increases MA, ZA, and VA, respectively. Each parameter individually contributes more than 45% to its corresponding advective feedback component, suggesting a robust dynamic adjustment of ocean current under historical forcing.

Therefore, we conclude that the changes in feedback terms primarily arise from the variations in the $\beta_{i}$ coefficients (i=h,ur,vr,wr). Here, we used the established dynamical framework to give a more comprehensive physical interpretation. One of the prominent structural changes in the tropical Pacific under historical forcing is the shoaling of the equatorial TH. In the reduced-gravity framework, this shoaling has direct implications for the dynamical response of the ocean to surface wind forcing. Because wind stress primarily acts on the upper layer, and as this upper layer becomes thinner, momentum per unit depth increases, thereby intensifying the sensitivity of ocean to atmospheric forcing (*22*). As a result, the feedback coefficients $\beta_{i}$ are expected to increase as the TH depth H decreases. This relationship can be denoted as $\Delta \beta_{i}\propto -\Delta H$ (*23*). Our analysis provides clear support for this relationship across CMIP6 models: Models exhibiting stronger TH shoaling also exhibit larger increases in the corresponding $\beta_{i}$ values (Fig. 3, K to N). As for $F_{1}$ and its components, we find no notable changes (fig. S11).

A direct dynamical consequence of an increased ENSO growth rate is an amplification of ENSO variability. This is consistent with both observational and model studies (*6*). A larger amplitude naturally enhances the signal-to-noise ratio of ENSO events, making them more predictable. This link between ENSO amplitude and predictability has been previously documented in both observational and modeling studies (*24*–*26*). To substantiate this connection in our framework, we find that intermodel variations in the change of ENSO growth rate (*R*) are positively correlated with changes in ENSO amplitude across CMIP6 models (fig. S12). This further supports our conclusion that enhanced positive feedbacks under historical forcing not only increase the growth rate but also amplify ENSO amplitude, thereby improving forecast skill.

## DISCUSSION

**Fig. 4.**
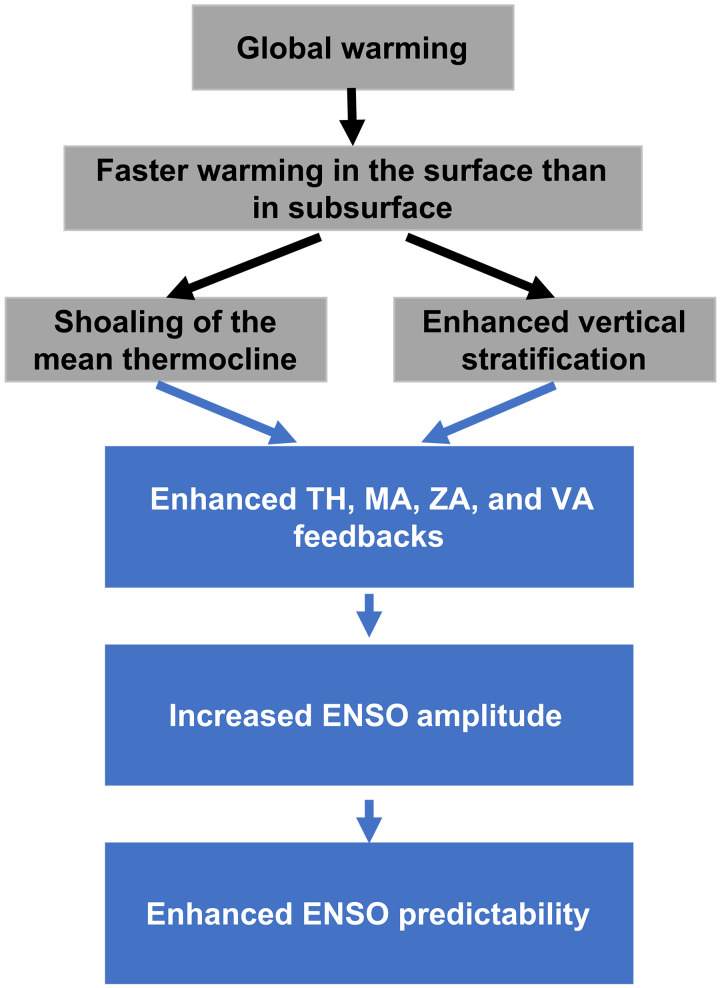
Mechanism underlying enhanced ENSO predictability under historical forcing. Under historical greenhouse-gas forcing, the tropical Pacific experiences stronger surface warming relative to the subsurface, leading to a systematically shallower mean TH and increased stratification in the equatorial upper ocean. This shoaling TH amplifies the efficiency of ocean-atmosphere coupling by intensifying three TH, ZA, and VA feedback. Such enhanced feedback boost intrinsic ENSO growth rate explains the enhanced ENSO predictability under historical forcing.

In this study, using a CNN model combined with a leave-one-out model strategy, we identify a robust enhancement of ENSO predictability under historical forcing. The key ocean-atmosphere processes contributing to this enhancement are summarized in Fig. 4. Under historical forcing, the equatorial Pacific experiences enhanced mean vertical stratification, primarily driven by faster warming of the upper ocean relative to deeper layers. This differential warming arises from greenhouse gas–induced radiative forcing and surface freshening associated with intensified precipitation. These changes also lead to a shoaling of the TH. Together, these changes amplify the sensitivity of the surface mixed layer to wind forcing. The enhancement of this positive feedback contributes to a stronger ENSO growth rate, lift the ENSO amplitude, and ultimately increase the ENSO predictability.

While this study mainly concentrates on the differences between the preindustrial and historical periods, the results can also be understood within the wider framework of previous work that examined ENSO predictability under SSP5-8.5 scenarios (*9*). A key distinction lies in the choice of reference climate. When referenced to the preindustrial control, both the historical period and the SSP5-8.5 scenario (fig. S13) exhibit enhanced ENSO predictability, consistent with a strengthening of dynamical (advection-related) feedbacks relative to the preindustrial climate. In contrast, studies that evaluate SSP5-8.5 relative to the historical baseline emphasize a different aspect of the climate response, namely the emergence of stronger thermodynamic damping associated with surface warming, which can reduce predictability despite continued dynamical enhancement (*9*). We repeated the analysis shown in Fig. 1 for the SSP5-8.5 scenario, those results are shown in fig. S13. Compared to PiControl, predictability under SSP5-8.5 remains enhanced, but with reduced model agreement (61.3%) compared with the historical experiment (83.9%). These results indicate that the predictability response evolves continuously with increasing forcing. ENSO variability is known to change under a warming climate (*2*, *6*), and the transition from enhanced to reduced predictability likely reflects the shifting balance between dynamical growth and thermodynamic damping. Determining when and under which forcing conditions thermodynamic processes become dominant is an important question, but a detailed investigation of this transition needs a further study.

Our results also demonstrate how deep learning–based climate emulators can be used as an efficient framework for investigating climate predictability. Building on existing emulator approaches trained on conventional climate simulations (*27*), this method avoids the high computational cost of large forecast experiments with traditional coupled general circulation models (CGCMs). The robust behavior shared across multi-CNN reforecast experiments yields a useful signal that, when combined with physical understanding, advances knowledge of Earth system (*28*). From the perspective of ENSO predictability, our results show how evolving background climate conditions modulate predictability, contributing to a more comprehensive understanding of ENSO in a warming climate.

## MATERIALS AND METHODS

### Reanalysis and CMIP6 models

Monthly reanalysis SST and SSH data were obtained from the European Centre for Medium-Range Forecasts Ocean Reanalysis System 5 (ORAS5, 1980 to 2015) and Simple Ocean Data Assimilation version 2.2.4 (SODA, 1900 to 1979) (*29*). The CMIP6 multimodel ensemble includes monthly fields of SST, SSH, zonal, meridional and vertical current, zonal wind stress, and downward net heat flux. We selected the models under both preindustrial (piControl) and historical forcing, each spanning 115 years (see table S1). Three models were excluded because their El Niño peak state fall outside the Niño 3.4 region, which is not consistent with the observation (fig. S1). To give a more compelling physical basis for model selection and to ensure that the selected models realistically represent ENSO behavior, we adopt established ENSO evaluation metric used in previous studies (*30*). First, we quantify three important feedbacks using linear regressions: (i) SST-Taux feedback, computed by regressing zonal wind-stress anomalies (Taux) in the Niño4 region onto SSTA in the Niño3 region; (ii) Taux-SSH feedback, computed by regressing SSHA in the Niño3 region onto Taux in the Niño4 region; and (iii) SST-SSH feedback, computed by regressing SSHA onto SSTA within the Niño3 region. Second, we consider ENSO pattern, amplitude, and seasonality. The ENSO pattern is defined as the root mean square error of SSTA over the equatorial Pacific ($150^{\circ }$E–$90^{\circ }$W) during boreal winter, relative to observations. ENSO amplitude is defined as the SD of the Niño 3.4 index, while ENSO seasonality is measured as the ratio of winter to spring SD of Niño 3.4 region SSTA. On the basis of the rankings reported in (*30*), we select 15 CMIP6 models that perform better than average across these metrics from our model ensemble and repeat the analysis shown in Fig. 1 for this subset. The corresponding results (figs. S3 and S4) are consistent with the full-model ensemble, showing that the ENSO predictability is enhanced under historical forcing.

To reduce the computational demands of our CNN model, all the SST and SSH data were interpolated onto a $5^{\circ }\times 5^{\circ }$ grid. For the subsequent analyses, we increased the spatial resolution to $1^{\circ }\times 1^{\circ }$ and performed analysis on the last 35 years data. The seasonal cycle was removed from the data based on the monthly climatology computed over their respective period. We only linear detrend the historical forcing experiments as there is no notable warming trend in piControl runs.

### The evaluation metrics

The model prediction skill is quantified using the ACC, which is defined as

ACC=<Fi,Oi><Fi,Fi><Oi,Oi> (1)

where $F_{i}$ is the ensemble mean forecast anomaly for forecast month and $O_{i}$ is the corresponding truth. The < > denotes the variance over all the months in verifying time series. Then, the prediction skill r(m,t) (ACC in this study) can be written as the function of initial months m and lag months t. The maximum gradient for initial month m is

$$G\left(m\right)=\underset{t}{\text{max}}\left[\frac{r\left(m,t-1\right)-r\left(m,t+1\right)}{2}\right]$$ (2)

The strength of this SPB can be defined as

$${\mathrm{SPB}}_{1}=\sum_{m=1}^{12}G\left(m\right)$$ (3)

As different models may exhibit different timing of predictability barrier (*31*), to explicitly represent the prediction barrier in spring, we also calculate the decay rate of the ACC in spring (*32*, *33*) and summarized them as in Eq. 3, defined as the $\mathrm{SP}B_{2}$ in this study. The results are presented in fig. S5.

### CNN model and experiment design

We use the same architecture as in a previous study (*10*), with only SST and SSH fields from CMIP6 for training. The CNN forecast model has three convolutional layers and two max-pooling layers between the layers. Then, a fully connected layer is linked to the output of the convolutional layers to get the final output. For simplicity, we set the number of convolutional filters and neurons in the fully connected layer as 50. The input data are the monthly SST and SSH fields from the preceding 12 months and predicts the Niño 3.4 index for the subsequent 24 months. The batch size is set to 300 and root mean square error as loss function. The model is trained using Adam optimizer, the initial learning rate was set to $3\times 10^{-5}$ and gradually annealed to 0 following the cosine schedule with 800 epochs.

To assess the ENSO predictability across the CMIP6 models (see table S1) under two forcings (piControl and historical), we use a leave-one-out model strategy. For each target model, we train a CNN model using data from the remaining 30 models. Each of these training models contributes 160-year data: the 1900 to 1979 segment of its historical run plus the first 80 years of its piControl run. To avoid overfitting, we reserve the target model’s 160-year data as a validation set (1900 to 1979 from its historical run plus the first 80 years of its piControl run) and stop training if the validation loss increases for three consecutive epochs. After training, we apply the CNN to the withheld model’s output from 1980 to 2014 in its historical run and the final 35 years of its piControl run to predict its ENSO index. For example, to evaluate ENSO predictability in ACCESS-CM2, the CNN is trained on data from the other 30 models, and validation uses ACCESS-CM2’s historical run from 1900 to 1979 and piControl years 950 to 1029. Prediction is then performed for 1980 to 2014 in historical runs and 1030 to 1064 in piControl runs in ACCESS-CM2. By repeating this process for all 31 models, thereby quantifying each model’s intrinsic ENSO predictability under two different forcings. For the real-world prediction, we train the model with all the CMIP6 historical run data and validate with the SODA reanalysis (1900 to 1979). All the observation-based results are calculated with the ORAS5 dataset.

### ROM and ensemble prediction experiment

Previous studies formulated the linear ENSO dynamics into two prognostic equations for eastern equatorial Pacific SSTA ($T_{E}$) and warm pool TH depth anomaly ($h_{w}$) (*18*), which is known as ROM

$$\frac{dT_{E}}{\mathrm{dt}}={\mathrm{RT}}_{E}+F_{1}h_{w}+\xi$$ (4)

$$\frac{dh_{w}}{\mathrm{dt}}=-{\varepsilon h}_{w}-F_{2}T_{E}$$ (5)

Here, the ξ is white noise forcing. We perform the ensemble forecasts using ROM to understand ENSO predictability. We first spin-up ROM for 10 years and then integrated for 300 years to generate the “truth” outputs. The integration time step is 0.01 months. From this, truth 20-member ensemble members are initialized by adding random perturbations to $T_{E}$, with amplitude 0.1$\sigma \left(T_{E}\right)$. Here, $\sigma \left(T_{E}\right)$ is the SD of $T_{E}$. Each ensemble member is forecast for 300 years with a fixed 12-month lead time. Predictability in ROM is defined as the averaged ACC over lead 1 to 12 months. Different lead time ranges are chosen for the ROM and CNN models to account for their differing predictive capacities, previous studies have shown that deep learning models deliver substantially higher prediction skill than CGCM-based ENSO forecasts, particularly at intermediate and longer lead times (from approximately 12 months onward) (*20*, *34*). That is why we use the 1- to 24-month average for CNN to characterize ENSO predictability across seasonal to interannual timescales. However, as the impact of model errors on predictions will increase, when the prediction time is too long the model error will dominant the model skill (*35*, *36*), we examined different choices of lead time and found that our results are insensitive to the selection of different lead time for the CNN model (fig. S2).

In ROM prediction experiment, we assess the intrinsic predictability of ENSO dynamics using ROM. All the model parameters are derived from CMIP6 model outputs, and the Niño3.4 index is predicted accordingly, allowing us to evaluate the predictability of 31 CMIP6 models under historical and preindustrial forcing. Although absolute prediction skill of ROM is lower than that of CNN, our focus is on whether the change in ROM skill is comparable to the corresponding change in CNN skill. Matching these changes allows us to interpret the CNN skill changes in terms of the underlying ROM dynamics.

In the four perfect model experiments, the forecast model uses the same parameter set that produced truth. The truth is generated by varying one parameter while holding the other three at their default ($R=-0.055\;\text{mont}h^{-1}$, $F_{1}=0.15^{\circ }C\cdot m^{-1}\cdot \text{mont}h^{-1}$, $\varepsilon =0.14\;{\text{month}}^{-1}$, and $F_{2}=2.5\;m\cdot^{\circ }C\cdot \text{mont}h^{-1}$). The four experiments therefore isolate the influence of each parameter on ENSO predictability. The default *R* is the average of the ensemble mean values estimated from the two forcing experiments (−0.07 and −0.04 ${\text{month}}^{-1}$, respectively). The results are shown in Fig. 2B and figs. S6 and S8.

The single imperfect model experiment, we predict the truth value with a biased *R* parameter. Both the historical truth (*R* = −0.04 ${\text{month}}^{-1}$) and the piControl truth (*R* = −0.07 $\text{mont}h^{-1}$) are forecast using *R* = −0.07 $\text{mont}h^{-1}$, with all other parameters at their defaults. Therefore, for historical forecasts, the prediction model is imperfect. The results are shown in fig. S9. This test demonstrates that our predictability results are insensitive to errors in the forecast-model dynamics, confirming their robustness.

### Ocean-atmosphere feedbacks

On the basis of heat budget analysis, the growth rate R (also known as BJ stability index) of $T_{E}$ can also be cast as (*17*)

$$\begin{array}{c} R=\mu_{a}\beta_{h}a_{h}\gamma \frac{<M\left(\bar{w}\right)\bar{w}>}{H_{m}}-\left[\frac{<\bar{u}>}{L_{x}}+\frac{<-2y\bar{v}>}{L_{y}^{2}}+\gamma \frac{<M\left(\bar{w}\right)\bar{w}>}{H_{m}}\right]\\ +\left(\mu_{a}\beta_{\mathrm{ur}}+\mu_{a}^{*}\beta_{\mathrm{ul}}\right)<-\partial_{x}\bar{T}>+\left(\mu_{a}\beta_{\mathrm{vr}}+\mu_{a}^{*}\beta_{\mathrm{vl}}\right)<-\partial_{y}\bar{T}{>}_{A}+\\ \left(\mu_{a}\beta_{\mathrm{wr}}+\mu_{a}^{*}\beta_{\mathrm{wl}}\right)<M\left(\bar{w}\right)\partial_{z}\bar{T}>-\alpha \end{array}$$ (6)

Here, we mainly focused on the important feedback relevant to this study; the detailed description can be seen in (*17*). The term on the right side of Eq. 6 represents TH feedback, DD, ZA feedback, MA feedback, VA feedback, and TD, respectively. $H_{m}$ represent the mixed layer depth, which is 50 m in this study. u,v,w indicate the zonal, meridional, and vertical velocity. The symbol overbar represents the climatology value, and the bracket denotes the volume average in the eastern Pacific box ($5^{\circ }S-5^{\circ }N,190^{\circ }W-90^{\circ }W$). $\mu_{a}$ is the central equatorial Pacific ($5^{\circ }S-5^{\circ }N,150^{\circ }E-130^{\circ }W$) anomalous zonal wind forcing response to eastern Pacific SSTA forcing. $\beta_{h}$, $\beta_{\mathrm{ur}}$, $\beta_{\mathrm{vr}}$, and $\beta_{\mathrm{wr}}$ represent the responses of zonal anomalous SSH gradient, three-dimensional current in the eastern Pacific to anomalous central equatorial wind forcing. $a_{h}$ denotes the subsurface (75 m in this study) temperature response SSHA in the eastern Pacific Ocean. $L_{x}$ and $L_{y}$ are the zonal and meridional scale of the equatorial Pacific Ocean, y is the distance from the Equator. γ is the vertical mixing efficiency coefficient, which is set to 0.75 in this study. All these responses are estimated with linear regression. The function M(x) is the Heaviside step function, is used to account for only the upwelling process. The $F_{1}$ can also be derived as

$$F_{1}=\beta_{\mathrm{uh}}<-\partial_{x}\bar{T}>+\beta_{\mathrm{vh}}<-\partial_{y}\bar{T}{>}_{A}-\beta_{\mathrm{wh}}<M\left(\bar{w}\right)\partial_{z}\bar{T}>+a_{h}\gamma \frac{<M\left(\bar{w}\right)\bar{w}>}{H_{m}}$$ (7)

The term on the right side of Eq. 7 represents ZA feedback, MA feedback, VA feedback, and TH feedback, respectively. $\beta_{\mathrm{uh}}$, $\beta_{\mathrm{vh}}$ and $\beta_{\mathrm{wh}}$ represent the responses of three-dimensional current in the eastern Pacific to anomalous western Pacific TH depth anomalies.

### Quantifying the contribution of parameters

To quantify the contribution of each factor in changes in a composite variable, we consider two sets of experiments. Let i index each individual sample. In a linear case, where $\varphi_{1}^{\left(i\right)}=x_{1}^{\left(i\right)}+y_{1}^{\left(i\right)}$ and $\varphi_{2}^{\left(i\right)}=x_{2}^{\left(i\right)}+y_{2}^{\left(i\right)}$, the change in φ for sample i is $\delta \varphi^{\left(i\right)}=\varphi_{2}^{\left(i\right)}-\varphi_{1}^{\left(i\right)}$. The contribution of x to $\delta \varphi^{\left(i\right)}$ is defined as $C\left[x^{\left(i\right)}\right]=x_{2}^{\left(i\right)}-x_{1}^{\left(i\right)}$, and its fractional contribution is

$$f\left[x^{\left(i\right)}\right]=\frac{\left|C\left[x^{\left(i\right)}\right]\right|}{\left|C\left[x^{\left(i\right)}\right]\right|+\left|C\left[y^{\left(i\right)}\right]\right|}$$ (8)

We use the absolute values to ensure numerical stability, especially in cases where the net contributions are small. In the nonlinear case such as $\varphi_{1}^{\left(i\right)}=x_{1}^{\left(i\right)}y_{1}^{\left(i\right)}z_{1}^{\left(i\right)}$, we use a first-order approximation. For example, the contribution associated with x is defined by

$$C\left[x^{\left(i\right)}\right]=\delta \left[x^{\left(i\right)}\right]\bar{y^{\left(i\right)}}\;\bar{z^{\left(i\right)}}$$ (9)

where $\bar{y^{\left(i\right)}}=\frac{y_{1}^{\left(i\right)}+y_{2}^{\left(i\right)}}{2}$ and $\bar{z^{\left(i\right)}}=\frac{z_{1}^{\left(i\right)}+z_{2}^{\left(i\right)}}{2}$ are the averages between the two experiments. Here, $\delta \left[x^{\left(i\right)}\right]$ denotes the difference between two experiments, here is $\left[x_{2}^{\left(i\right)}-x_{1}^{\left(i\right)}\right]$. As a concrete instance for the TH feedback in kth model, the contribution of $\mu_{a}$ is given by $C\left[\mu_{a}^{\left(k\right)}\right]=\gamma \delta \left[\mu_{a}^{\left(k\right)}\right]\;\bar{\beta_{h}^{\left(k\right)}}\;\bar{a_{h}^{\left(k\right)}}\;\bar{{\frac{<M\left(\bar{w}\right)\bar{w}>}{H_{m}}}^{\left(k\right)}}$, and the analogous terms are $C\left[\beta_{h}^{\left(k\right)}\right]=\gamma \delta \left[\beta_{h}^{\left(k\right)}\right]\bar{\mu_{a}^{\left(k\right)}}\;\bar{a_{h}^{\left(k\right)}}\;\bar{{\frac{<M\left(\bar{w}\right)\bar{w}>}{H_{m}}}^{\left(k\right)}}$, $C\left[a_{h}^{\left(k\right)}\right]=\gamma \delta \left[a_{h}^{\left(k\right)}\right]\bar{\mu_{a}^{\left(k\right)}}\;\bar{\beta_{h}^{\left(k\right)}}\;\bar{{\frac{<M\left(\bar{w}\right)\bar{w}>}{H_{m}}}^{\left(k\right)}}$, $C\left[{\frac{<M\left(\bar{w}\right)\bar{w}>}{H_{m}}}^{\left(k\right)}\right]=\gamma \delta \left[{\frac{<M\left(\bar{w}\right)\bar{w}>}{H_{m}}}^{\left(k\right)}\right]\bar{\mu_{a}^{\left(k\right)}}\;\bar{a_{h}^{\left(k\right)}}\;\bar{\beta_{h}^{\left(k\right)}}$. The overbars indicate the average between historical and preindustrial forcing, and δ(·) means the historical minus preindustrial. Last, the fractional contribution of each term is computed as the absolute, weighted mean of its contribution.

### Bootstrap test

The bootstrap method was used to estimate the confidence intervals. For each group, data were resampled with replacement more than 10,000 iterations, where each bootstrap sample preserved the original group size. During this process, individual observations could be selected multiple times, thereby simulating the sampling variability of the target statistic (e.g., mean and SD). If the difference in mean values between two groups is greater than the sum of the SD values, the difference is considered statistically significant at the 95% confidence level.
