# Supplementary material for: Deep learning reveals enhanced ENSO predictability under historical anthropogenic forcing
Source: Sci Adv. 2026 Jun 10;12(24):eaec9518. doi: 10.1126/sciadv.aec9518 (PMC13251828; doi:10.1126/sciadv.aec9518)
Supplement: Supplementary file 1 — Figs. S1 to S13 Table S1 [file sciadv.aec9518_sm.pdf]

Science Advances
AAAS

Supplementary Materials for

Deep learning reveals enhanced ENSO predictability under historical anthropogenic forcing

Zikuan Lin *et al.*

Corresponding author: Yishuai Jin, jinyishuai@126.com

Sci. Adv. **12**, eaec9518 (2026)
DOI: 10.1126/sciadv.aec9518

This PDF file includes:

Figs. S1 to S13
Table S1

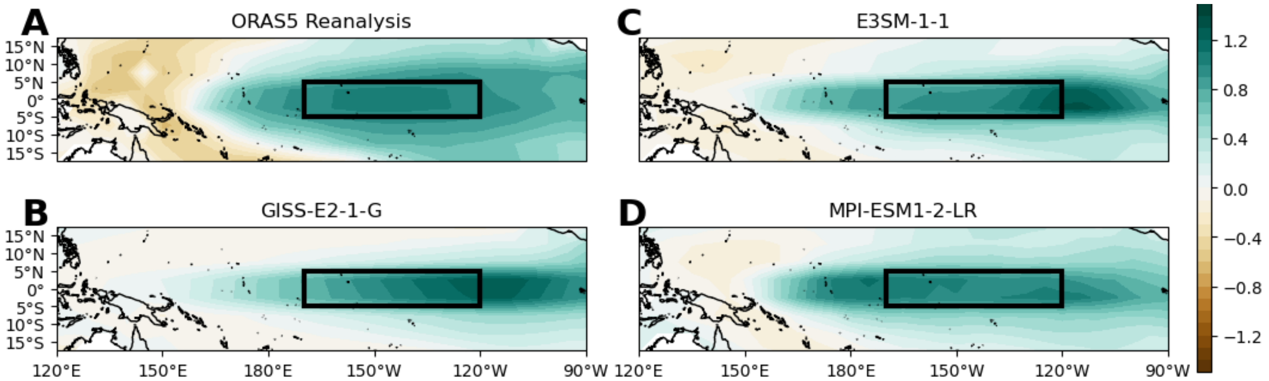

Fig. S1 Equatorial Pacific Sea surface temperature anomalies (SSTA) during El Niño peak phase from 1980 to 2014. Spatial SSTA regressed onto the respective Niño 3.4 index for (**A**) ORAS5 reanalysis, (**B**) GISS-E2-1-G, (**C**) E3SM-1-1 and (**D**) MPI-ESM1-2-LR. The Niño 3.4 region is marked by black boxes.

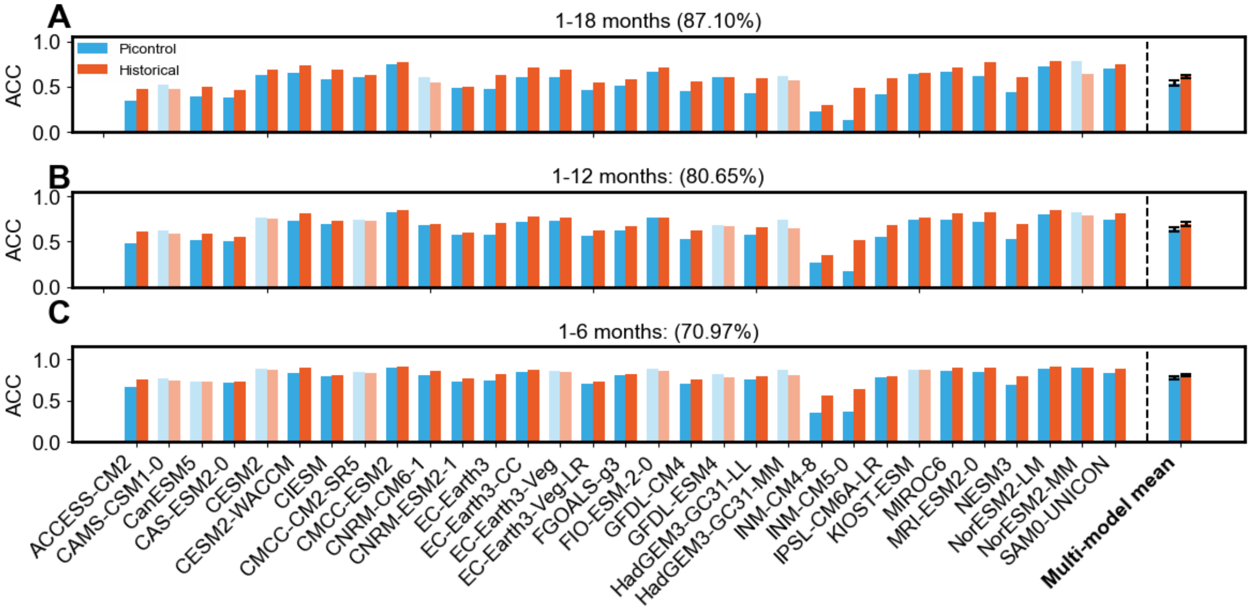

Fig. S2. CNN based estimation of ENSO predictability under pre-industrial and historical forcing, averaged over averaged from different lead times ranges. Same as Fig 1, but expressed as the ACC over averaged lead times of (**A**)1 to 18 months, (**B**) 1 to 12 months, (**C**) 1 to 6 months. The model consensus is shown in each panel title.

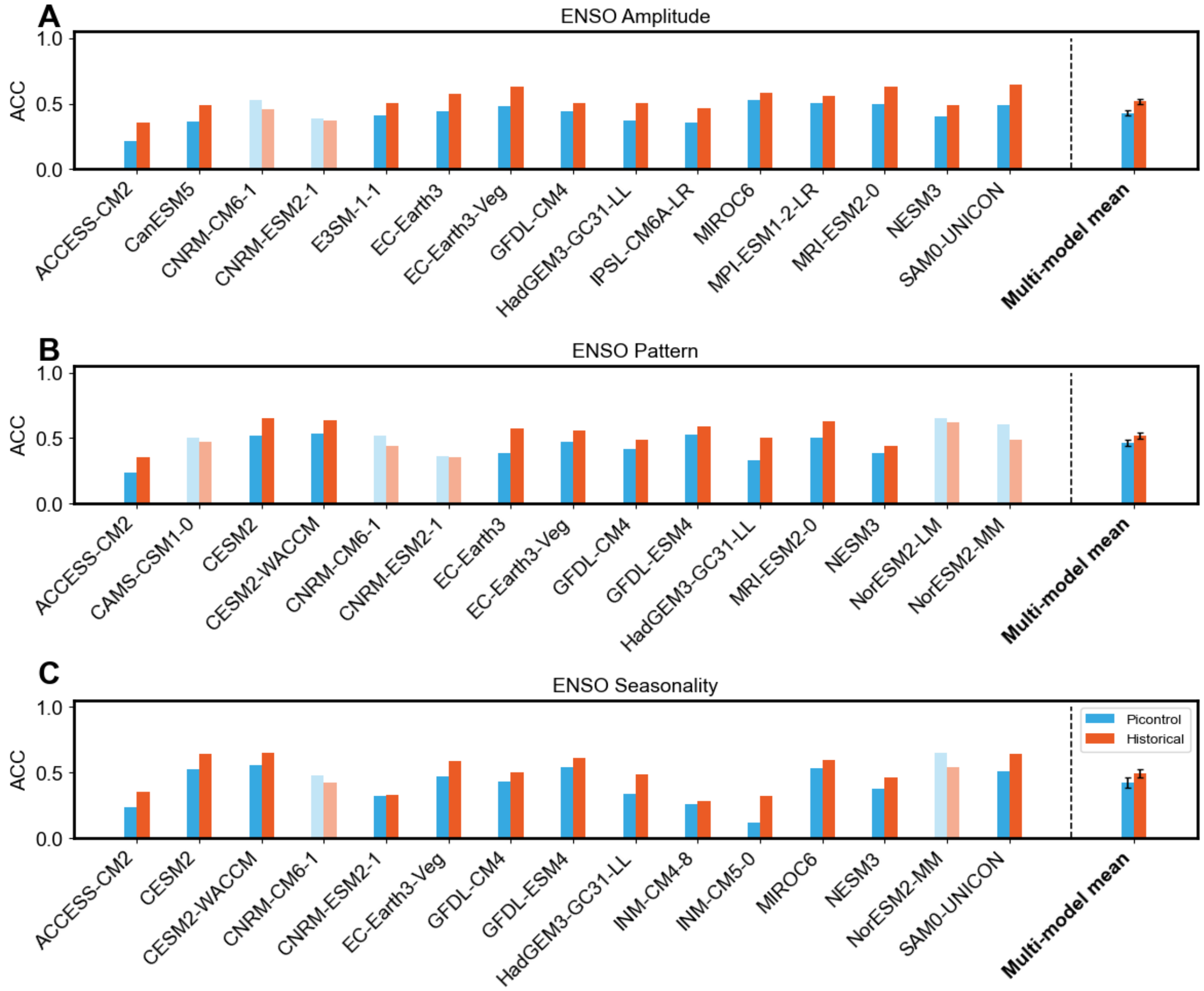

Fig. S3. CNN based estimation of ENSO predictability under pre-industrial and historical forcing, using the top 15 models selected according to different evaluation metrics. Model selection is based on (**A**) ENSO amplitude, (**B**) ENSO pattern, and (**C**) ENSO seasonality. Error bars indicate one standard deviation, computed via bootstrap resampling method. Models showing a trend opposite to the multi-model mean are displayed in lighted colors

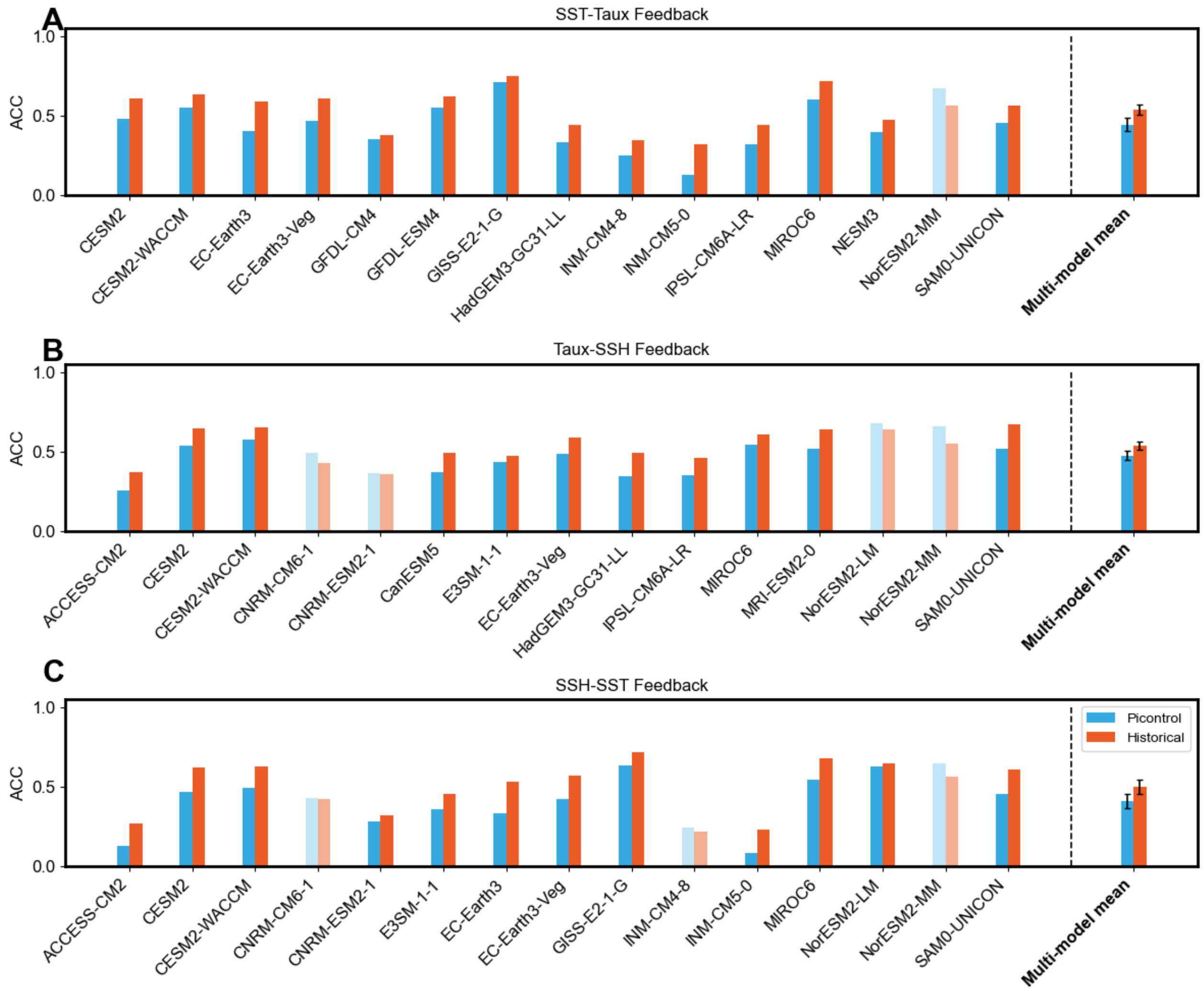

Fig. S4. CNN based estimation of ENSO predictability under pre-industrial and historical forcing, using the top 15 models selected according to different evaluation metrics. Model selection is based on (**A**) SST-Taux feedback, (**B**) Taux-SSH feedback, and (**C**) SSH-SST feedback. Error bars indicate one standard deviation, computed via bootstrap resampling method. Models showing a trend opposite to the multi-model mean are displayed in lighted colors

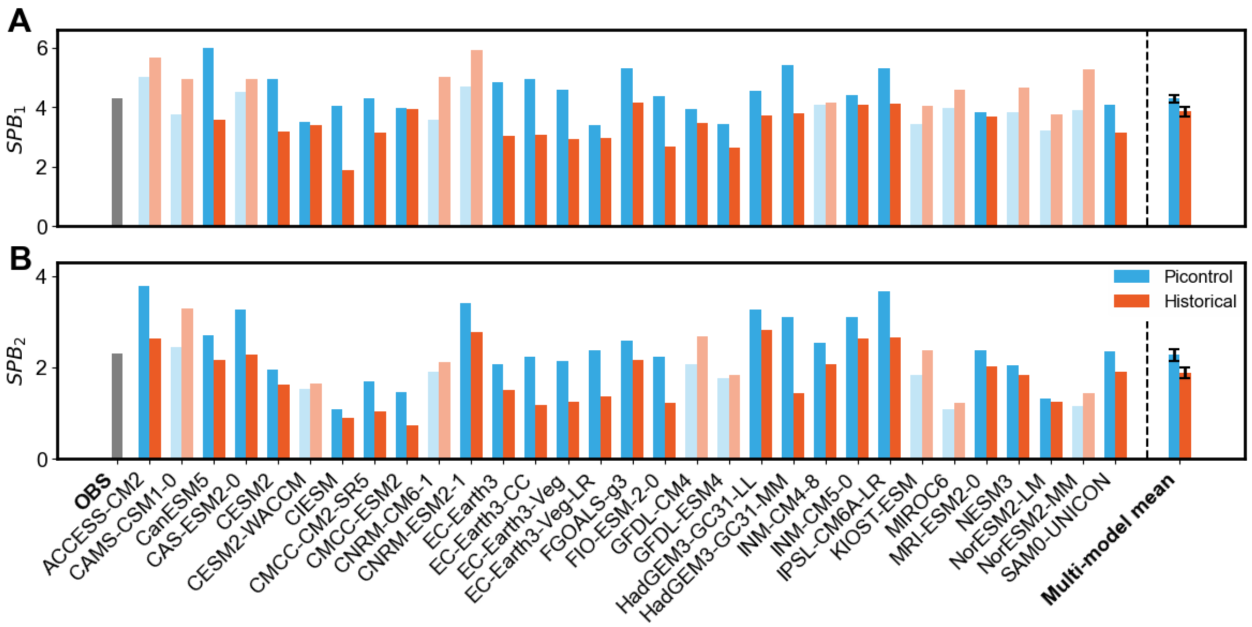

Fig. S5. CNN based estimation of spring predictability barrier (SPB) across CMIP6 models. (**A**) SPB strength defined by the maximum skill decay rate across all months. (**B**) SPB strength defined by the decay rate during spring. Error bars indicate one standard deviation, computed via bootstrap resampling method. Models showing a trend opposite to the multi-model mean are displayed in lighted colors

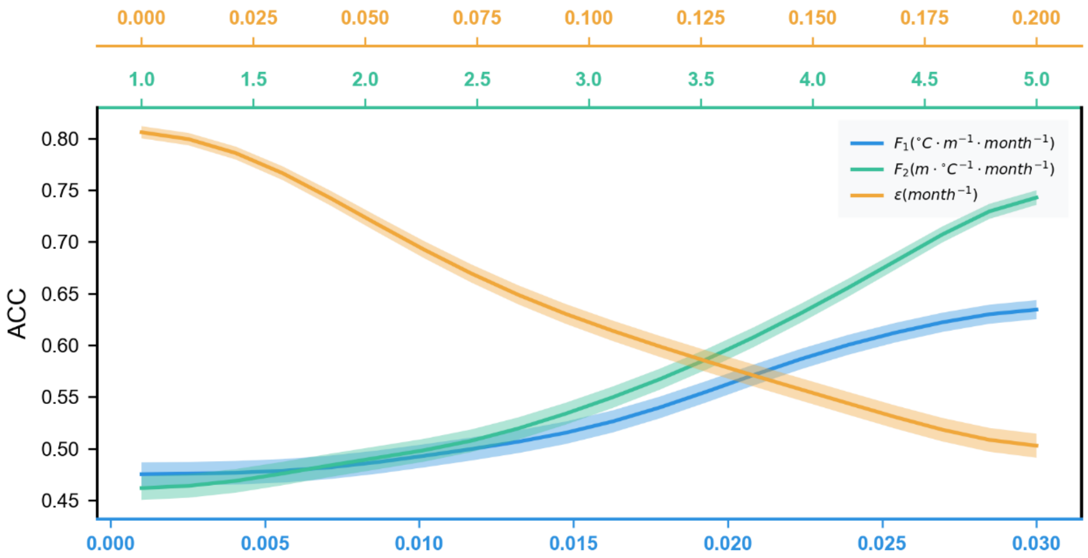

Fig. S6. Influence of parameters in recharge oscillator on ENSO predictability. Same as Fig. 2 (B), but for $\boldsymbol{F_1}$ (blue), $\boldsymbol{F_2}$ (green) and ε (orange). The detailed parameter setting, see Methods. The shading indicates one standard deviation, computed via bootstrap resampling method.

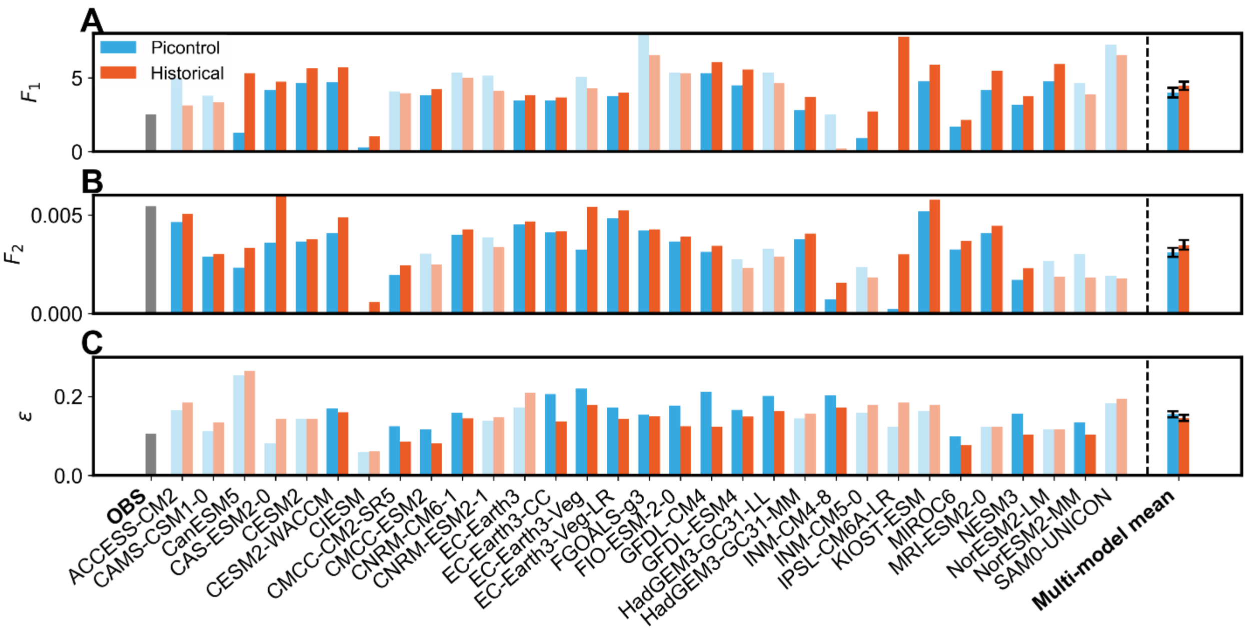

Fig. S7. Changes of parameters in recharge oscillator under pre-industrial versus historical forcing. Same as Fig. 2 (C), but for (**A**) $F_1(\,^{\circ}C \cdot m^{-1} \cdot month^{-1})$, (**B**) $F_2(m \cdot \,^{\circ}C^{-1} \cdot month^{-1})$ and (**C**) $\varepsilon(month^{-1})$. Error bars indicate one standard deviation, computed via bootstrap resampling method. Models showing a trend opposite to the multi-model mean are displayed in lighted colors.

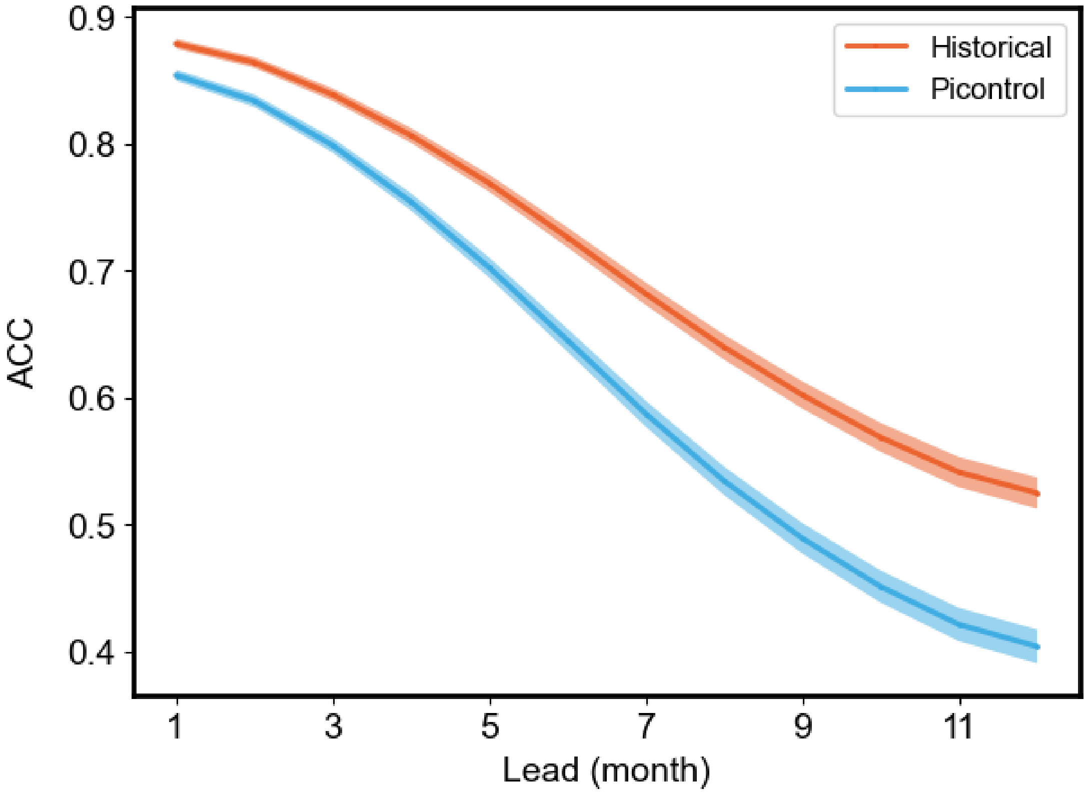

Fig. S8. Influence of ENSO growth rate on ENSO predictability under recharge oscillator framework. Prediction skill of ROM with different lead time, using the parameters marked by stars in **Fig. 2 (B)** for each forcing scenario. The shading indicates one standard deviation, computed via bootstrap resampling method.

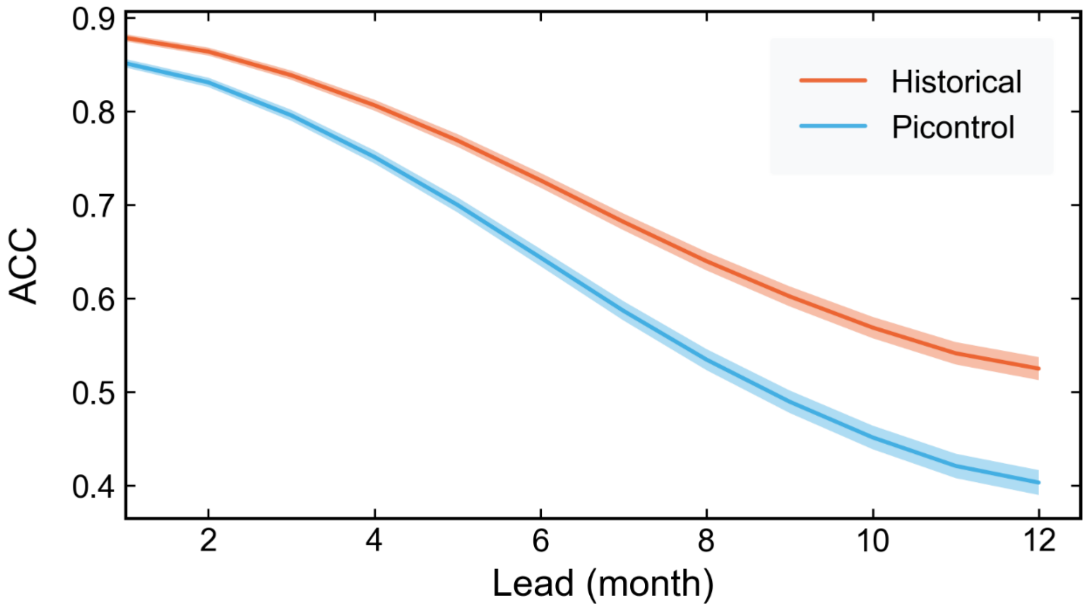

Fig. S9. Influence of ENSO growth rate on ENSO predictability under recharge oscillator framework. Same as **Fig. S8**, but in the Historical experiment, we set the model with "imperfect" parameter (R = -0.07). The shading indicates one standard deviation, computed via bootstrap resampling method.

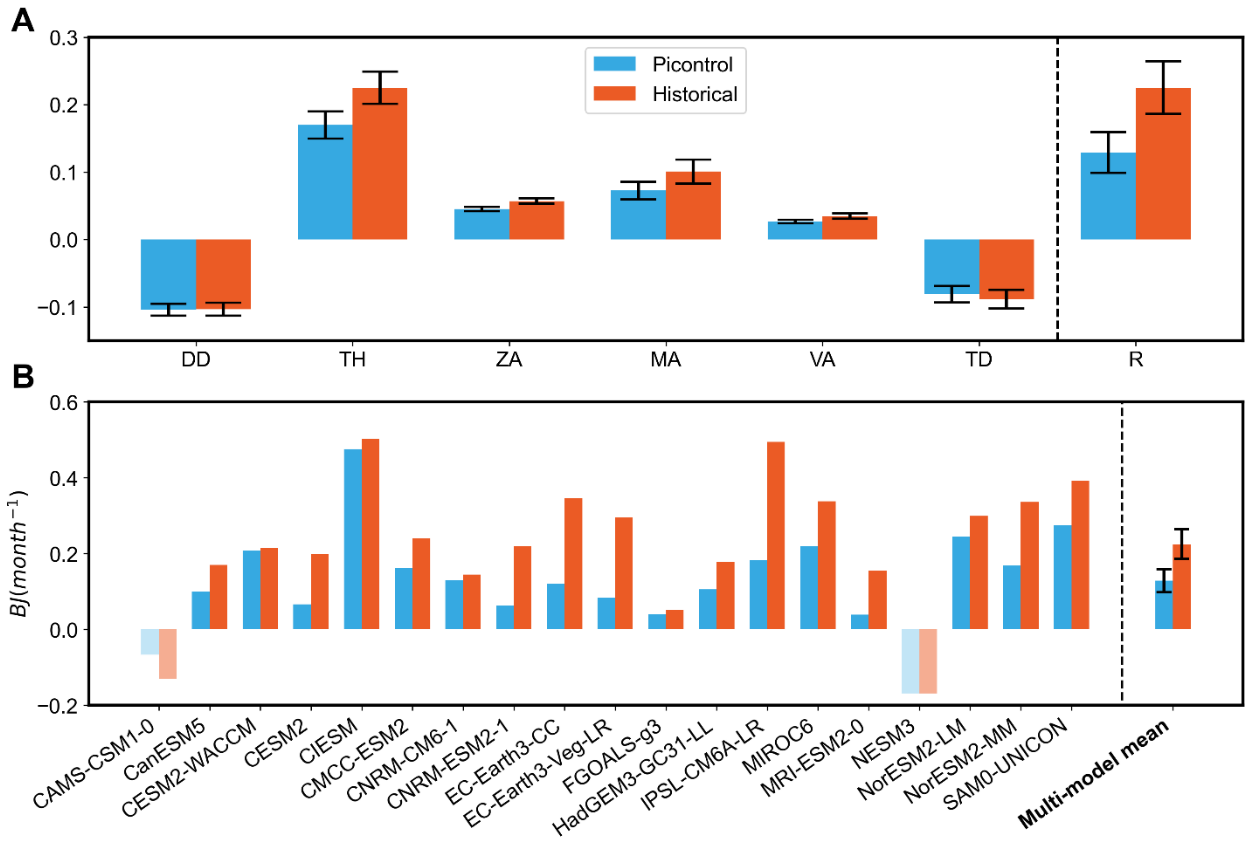

Fig. S10. Bjerknes index across different CMIP6 models and its components across CMIP6 models. (A) Multi-model ensemble mean of Bjerknes (BJ) index and its components: DD for dynamical damping, TH for thermocline feedback, ZA for zonal advective feedback, MA for meridional advective feedback, VA for vertical upwelling feedback, TD for thermal damping. (**B**) BJ index estimated from each CMIP6 models. Error bars denote one standard deviation, computed via bootstrap resampling method Models showing a trend opposite to the multi-model mean are displayed in lighted colors.

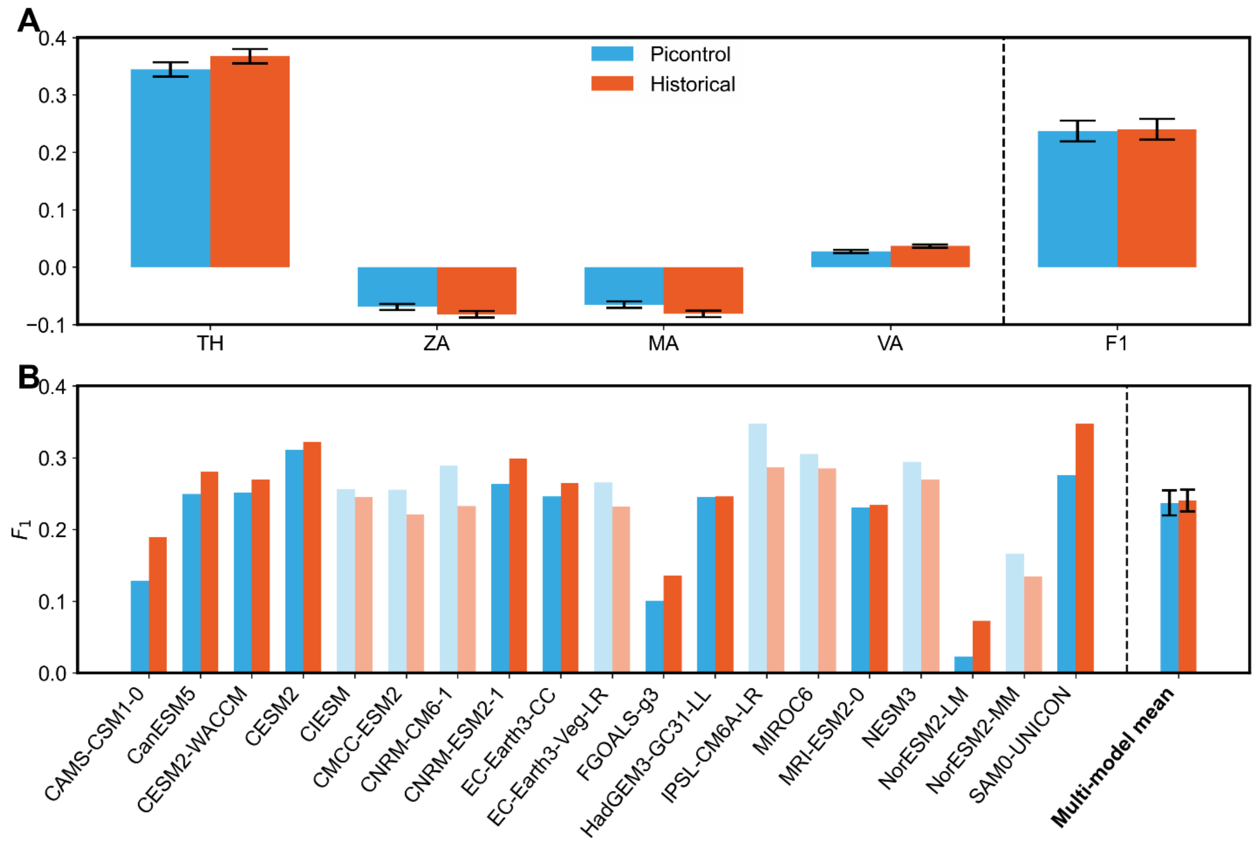

Fig. S11. F_1 **across different CMIP6 models and its components.** (**A**) Same as **Fig. S10A**, but for F_1. (**B**) Same as **Fig. S10B**, but for F_1. Error bars indicate one standard deviation, computed via bootstrap resampling method. Models showing a trend opposite to the multi-model mean are displayed in lighted colors.

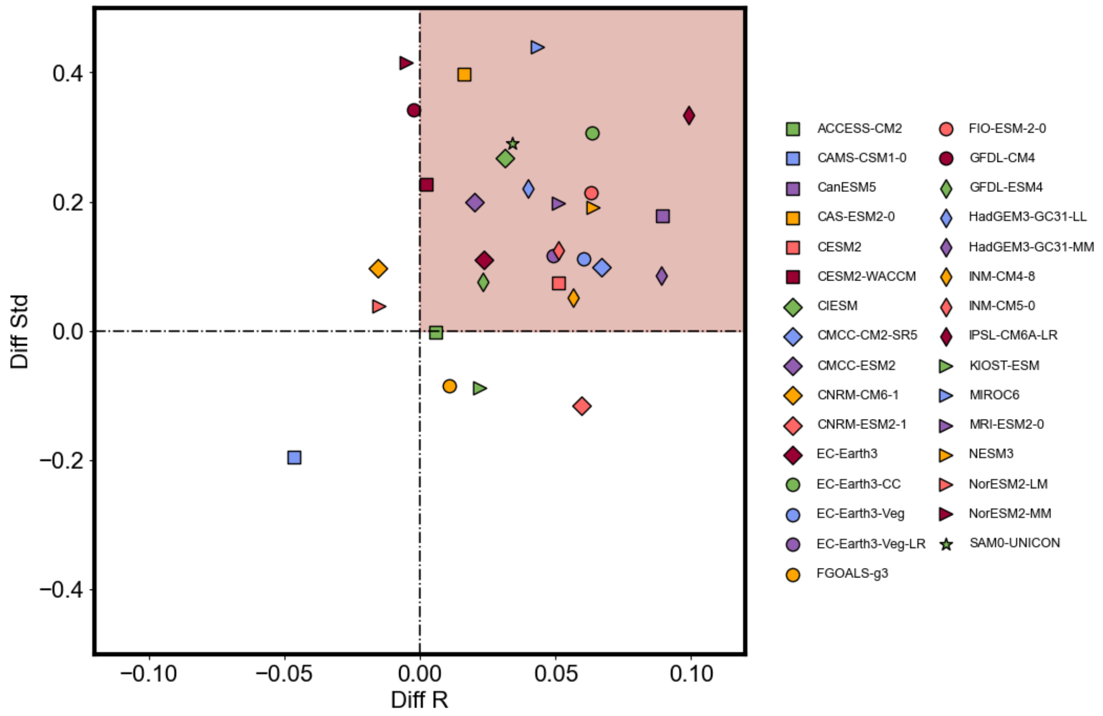

Fig. S12. Changes in ENSO growth rate versus ENSO amplitude across CMIP6 models. Each marker represents a CMIP6 model, as labeled on the right. Changes refers to historical minus PiControl.

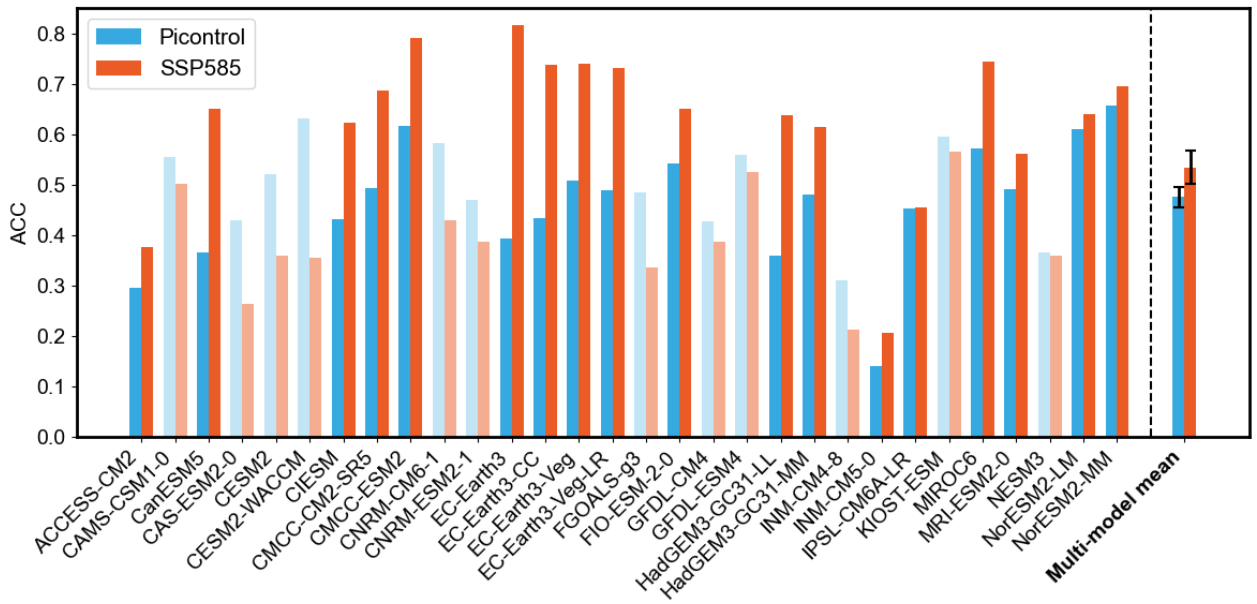

Fig. S13. CNN based estimation of ENSO predictability under pre-industrial and SSP5-8.5 forcing, averaged over averaged from different lead times ranges. Same as Fig 1, for the comparison between PiControl and SSP5-8.5.

Table S1. The Coupled Model Intercomparison Project phase 6 (CMIP6) models and variables

Model number	Model Name	Ensemble member	PiControl	Historical	Variables
1	ACCESS-CM2	r1i1p1f1	0950-1064	1900-2014	tos, zos
2	CAMS-CSM1-0	r1i1p1f1	3150-3264	1900-2014	tos, zos, hfds, tauuo, thetao, uo, vo, wo
3	CanESM5	r1i1p1f1	5201-5315	1900-2014	tos, zos, hfds, tauuo, thetao, uo, vo, wo
4	CAS-ESM2-0	r1i1p1f1	0001-0115	1900-2014	tos, zos
5	CESM2	r1i1p1f1	0001-0115	1900-2014	tos, zos, hfds, tauuo, thetao, uo, vo, wo
6	CESM2-WACCM	r1i1p1f1	0001-0115	1900-2014	tos, zos, hfds, tauuo, thetao, uo, vo, wo
7	CIESM	r1i1p1f1	0151-0265	1900-2014	tos, zos, hfds, tauuo, thetao, uo, vo, wo
8	CMCC-CM2-SR5	r1i1p1f1	1850-1964	1900-2014	tos, zos
9	CMCC-ESM2	r1i1p1f1	1850-1964	1900-2014	tos, zos, hfds, tauuo, thetao, uo, vo, wo
10	CNRM-CM6-1	r1i1p1f2	1850-1964	1900-2014	tos, zos, hfds, tauuo, thetao, uo, vo, wo
11	CNRM-ESM2-1	r1i1p1f2	1850-1964	1900-2014	tos, zos, hfds, tauuo, thetao, uo, vo, wo
12	E3SM-1-1	r1i1p1f1	1850-1964	1900-2014	tos, zos
13	EC-Earth3	r1i1p1f1	2259-2373	1900-2014	tos, zos,
14	EC-Earth3-CC	r1i1p1f1	1850-1964	1900-2014	tos, zos, hfds, tauuo, thetao, uo, vo, wo
15	EC-Earth3-Veg	r1i1p1f1	1850-1964	1900-2014	tos, zos
16	EC-Earth3-Veg-LR	r1i1p1f1	2300-2414	1900-2014	tos, zos, hfds, tauuo, thetao, uo, vo, wo
17	FGOALS-g3	r1i1p1f1	0700-0814	1900-2014	tos, zos, hfds, tauuo, thetao, uo, vo, wo
18	FIO-ESM-2-0	r1i1p1f1	0301-0415	1900-2014	tos, zos
19	GFDL-CM4	r1i1p1f1	0151-0265	1900-2014	tos, zos
20	GFDL-ESM4	r1i1p1f1	0001-0115	1900-2014	tos, zos
21	GISS-E2-1-G	r1i1p1f1	4150-4264	1900-2014	tos, zos
22	HadGEM3-GC31-LL	r1i1p1f1	1850-1964	1900-2014	tos, zos, hfds, tauuo, thetao, uo, vo, wo
23	HadGEM3-GC31-MM	r1i1p1f1	1850-1964	1900-2014	tos, zos
24	INM-CM4-8	r1i1p1f1	1850-1964	1900-2014	tos, zos
25	INM-CM5-0	r1i1p1f1	1996-2110	1900-2014	tos, zos
26	IPSL-CM6A-LR	r1i2p1f1	1850-1964	1900-2014	tos, zos, hfds, tauuo, thetao, uo, vo, wo
27	KIOST-ESM	r1i1p1f1	2689-2803	1900-2014	tos, zos
28	MIROC6	r1i1p1f1	3200-3314	1900-2014	tos, zos, hfds, tauuo, thetao, uo, vo, wo
29	MPI-ESM1-2-LR	r1i1p1f1	1850-1964	1900-2014	tos, zos
30	MRI-ESM2-0	r1i1p1f1	1850-1964	1900-2014	tos, zos, hfds, tauuo, thetao, uo, vo, wo
31	NESM3	r1i1p1f1	1000-1114	1900-2014	tos, zos, hfds, tauuo, thetao, uo, vo, wo
32	NorESM2-LM	r1i1p1f1	1600-1714	1900-2014	tos, zos, hfds, tauuo, thetao, uo, vo, wo
33	NorESM2-MM	r1i1p1f1	1200-1314	1900-2014	tos, zos, hfds, tauuo, thetao, uo, vo, wo
34	SAM0-UNICON	r1i1p1f1	0001-0115	1900-2014	tos, zos, hfds, tauuo, thetao, uo, vo, wo
